# Supplementary material for: Emergency Department Visits and Summer Temperatures in Bologna, Northern Italy, 2010–2019: A Case-Crossover Study and Geographically Weighted Regression Methods
Source: Int J Environ Res Public Health. 2022 Nov 24;19(23):15592. doi: 10.3390/ijerph192315592 (PMC9736574; doi:10.3390/ijerph192315592)
Supplement: Supplementary file 1 [file ijerph-19-15592-s001.zip › ijerph-1998115-supplementary.pdf]

Supplementary material

Table S1. List of variables used in the case-crossover analysis and level of availability

**variable**	**Level available**
EDV (outcome)	Individual
Mean apparent Temperature (exposure)	Daily base
Age-class (potential effect modifier)	Individual
Sex (potential effect modifier)	Individual
Town-district (potential effect modifier)	Individual
Deprivation index (potential effect modifier)	Census block level
Microclimatic discomfort index (potential effect modifier)	Urban block level
Population density (time-variant)	Daily base

Table S2. Log-likelihood values by temperature cut-off points

Temperature cut-off (°C)	Log-likelihood
15	-385,532.06
16	-385,532.11
17	-385,532.15
18	-385,532.11
19	-385,532.10
20	-385,531.95
21	-385,531.96
22	-385,531.84
23	-385,531.77
24	-385,531.66
25	-385,531.58
26	-385,531.41
27	-385,531.42
28	-385,531.63
29	-385,531.73
30	-385,532.13
